# Extracting Fractional Vegetation Cover from Digital Photographs: A Comparison of In Situ, SamplePoint, and Image Classification Methods

**DOI:** 10.3390/s21217310

**Published:** 2021-11-03

**Authors:** Xiaolei Yu, Xulin Guo

**Affiliations:** Department of Geography and Planning, University of Saskatchewan, Kirk Hall, 117 Science Place, Saskatoon, SK S7N 5C8, Canada; yux051@mail.usask.ca

**Keywords:** fractional vegetation cover, SamplePoint, image classification, OBIA, image analysis, Northern Mixed Grasslands

## Abstract

Fractional vegetation cover is a key indicator of rangeland health. However, survey techniques such as line-point intercept transect, pin frame quadrats, and visual cover estimates can be time-consuming and are prone to subjective variations. For this reason, most studies only focus on overall vegetation cover, ignoring variation in live and dead fractions. In the arid regions of the Canadian prairies, grass cover is typically a mixture of green and senescent plant material, and it is essential to monitor both green and senescent vegetation fractional cover. In this study, we designed and built a camera stand to acquire the close-range photographs of rangeland fractional vegetation cover. Photographs were processed by four approaches: SamplePoint software, object-based image analysis (OBIA), unsupervised and supervised classifications to estimate the fractional cover of green vegetation, senescent vegetation, and background substrate. These estimates were compared to in situ surveys. Our results showed that the SamplePoint software is an effective alternative to field measurements, while the unsupervised classification lacked accuracy and consistency. The Object-based image classification performed better than other image classification methods. Overall, SamplePoint and OBIA produced mean values equivalent to those produced by in situ assessment. These findings suggest an unbiased, consistent, and expedient alternative to in situ grassland vegetation fractional cover estimation, which provides a permanent image record.

## 1. Introduction

The Fractional vegetation cover (FVC) is defined as the percentage of the ground surface covered by vegetation elements from the overhead perspective [1]. This metric describes vegetation quality and composition, which contributes to ecosystem change and control transpiration and photosynthesis among other terrestrial processes [2]. For this reason, analyses of fractional vegetation cover are widely used in land surface modelling [3], ecosystem monitoring [4], and natural resource management [5]. The ability to conduct systematic, accurate, and repeatable vegetation fractional cover estimation is a fundamental part of ecosystem biodiversity and function studies.

In arid and semiarid rangelands, live and senescent vegetation materials are often intermixed and difficult to discriminate [6]. These materials both play vital, but different, roles in ecological cycling and natural grassland conservation. Senescent vegetation consists largely of prostrate litter and standing dead grass, but like live vegetation, provides forage for grazers [7], alters the microclimate at a local scale [8], provides habitats to many species at risk [9], and is crucial in grassland fire dynamics [10,11]. Hence, the simultaneous estimation of both green and senescent vegetation covers is important to understand, manage and conserve arid and semi-arid rangelands.

Many efforts have been made to measure vegetation fractional cover in the field. Conventional methods of ground FVC estimation include the line-point intercept transect (LPIT) [12] and the Daubenmire cover class methods [13]. Both are labor-intensive and prone to overestimating standing vegetation cover due to field survey design [14].

At large spatial scales, satellite-based remote sensing can be effective in estimating FVC [15,16]. Various vegetation indices have been developed to estimate FVC in arid and semiarid grasslands [2,15], and spectral mixing analysis (SMA) has been applied to FVC modeling [17]. However, satellite remote sensing approaches need to be calibrated with in situ data to maintain accuracy and account for spatial–temporal heterogeneity [18,19]. FVC field measurement remains critical to provide a baseline for improving inversion algorithms and validating remote sensing products [20].

Close range photography has become a popular method to estimate FVC in the field. It offers a nondestructive approach that has the potential to be equally as, or more accurate, faster, and less biased than, in situ techniques [21,22,23,24]. Digital images taken of small plots processed using supervised [25] or unsupervised [26] classification, object-based image analysis (OBIA) [27], and rule-based decision or machine learning [28] have been successful in objectively quantifying the percent of vegetation cover in a range of environments. In concert, many tools have been developed to process close-range digital images, including SamplePoint [29], VegMeasure [30], and Canopeo [23]. However, most research has focused on estimating the live or green vegetation fractional cover, ignoring senescent vegetation, which is a significant component of arid and semiarid prairie grasslands.

In arid and semiarid regions, senescent plant materials include standing dead biomass, dormant grass, or litter from nondecomposed biomass. These are often intermixed with the green vegetation making it challenging to differentiate the senescent and green vegetation fractional cover from the background [6]. While a few studies have compared the results of different image-processing methods to estimate green, nongreen and senescent vegetation [6,31], the accuracy of different approaches involving close-range imagery has yet to be thoroughly evaluated. This is particularly the case under conditions where senescent and green vegetation are mixed in native grasslands.

The main objective of this research is to evaluate the accuracy and consistency of vegetation fractional cover extracted using different methods, including in situ assessment and image analysis. To fulfill this objective, we used a camera stand to acquire close-range digital photographs in Grassland National Park (GNP), Saskatchewan, a typical Northern Mixed Grassland region. The digital images were then analyzed with SamplePoint software for visual interpretation and with Environment for Visualizing Image (ENVI) software [32] for pixel-based classification and object-based image analysis (OBIA) to obtain the fractional cover of senescent and green vegetation. We used in situ assessment to quantify the fractional vegetation cover (FVC) as a reference. FVC estimates from image processing were compared to field measurements to corroborate links and consistency.

## 2. Materials and Methods

### 2.1. Study Area

Fieldwork was conducted in the west block of Grasslands National Park (GNP; 49° N, 107° W), located in the semiarid, mixed grassland ecoregion of southern Saskatchewan, Canada [33], from 28 June to 3 July 2018. GNP lies in a region with a mean annual temperature of 4.1 °C and total annual precipitation of 352.5 mm [34]. Almost half of the annual precipitation falls as rain during the spring growing season, followed by a long, dry summer [35], during which annual plants die and perennial herbaceous plants wither above ground while their below-ground parts persist [36]. Therefore, there is a high proportion of brown and grey senescent vegetation (nonphotosynthetic biomass, NPV) above ground in addition to green, photosynthetic vegetation (PV).

### 2.2. Field Experiment Design

**Figure 1:**
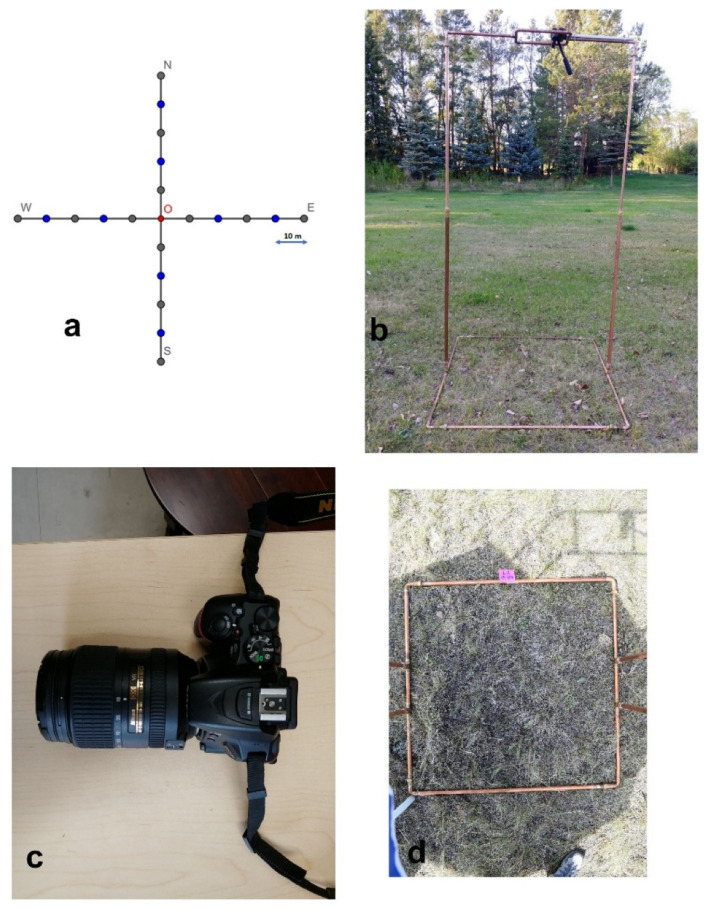
Illustration of field setup: (**a**) study-site sampling design, (**b**) camera stand, (**c**) NIKON D5500 camera, and (**d**) overhead photo of stand base frame.

We built a collapsible camera stand, two meters high with a one square meter (1 m × 1 m) base frame; each edge of the frame was marked by decile ticks to facilitate in situ measurement (Figure 1). A tripod with a pan-tilt head and independent axes and controls was attached to the top of the camera stand (Figure 1b). A NIKON D5500 camera with an AF-S DX 18–55 mm f/3.5–5.6 G lens was attached to the head (Figure 1c). The camera was held at a nadir position relative to the base frame by adjusting the pan-tilt head. An umbrella was used to shade the base above the frame. We used a remote release to control the shutter and mobile phone connected with the camera by Bluetooth to check photos. The camera was preset to user-shutter-priority mode with a maximum of 1/200 s shutter speed to avoid wind effects on the grass canopy imagery [37].

We surveyed nine sites across the study area’s dominant topographical features: valley, sloped, and upland grasslands. The distance between sites was at least 1.5 km to prevent the spatial autocorrelation [38]. At each site, two perpendicular transects, 100 m long and intersecting at their centers, were surveyed, oriented in the cardinal directions (Figure 1a). Five images were taken along each arm at 10 m intervals, excluding the center, thus, 20 images were recorded per site (Figure 1d). At each plot, we recorded the percentage cover of grass, forb, shrub, standing dead material, litter, lichen, moss, bare soil, and rock by visual assessment within each base frame after taking the photo of that frame. Percent cover was estimated to the nearest 5% for cover values ranging from 10 to 90% and the nearest 1% for values less than 10% and greater than 90% [39,40]. To limit the subjective bias, two people independently assessed the in situ cover and their interpretations were averaged. We sorted the original record of each plot fraction by summing up into PV (green grass, forb, shrub, green moss), NPV (standing dead material, litter), and BS (bare soil, rock, lichen).

### 2.3. Image Analysis

We estimated the fractional cover of PV, NPV, and BS from the digital image using four methods: (1) visual classification using SamplePoint software, (2) unsupervised image classification, (3) supervised image classification, and (4) object-based image analysis (OBIA). The unsupervised and supervised classifications as well as the OBIA were conducted using ENVI 5.5 (Harris Geospatial Inc. Broomfield, CO, USA) and ArcMap 10.6 (Esri Inc. Redlands, CA, USA).

SamplePoint is a popular software for visual inspection of ground cover in grassland and pasture research and management [29]. This software loads images listed by the user in an Excel spreadsheet, and systematically or randomly identifies and locates a user-defined number of sample points in the image. It then moves from one point to the next so that the user can classify each point visually [22]. We identified the 10 × 10 (100 in total) points systematically spread across each image for visual classification using the same nine categories as in the field assessment and grouped the results into PV, NPV, and BS for future analysis. Two independent assessments were performed on 180 images using the SamplePoint software and the results from the two assessments were averaged.

For the unsupervised classification, we followed methods described by Smith, Hill and Zhang [26,41]. Images were transferred from the original RGB (red, green, blue) color spectrum into HIS (hue, intensity, saturation) space. Images were divided into 14 classes using the ISODATA algorithm in ENVI 5.5 image analysis software. The original 14 classes were visually examined with reference to the original photos and were merged to derive PV, NPV, and BS. For the supervised classification, we used the maximum likelihood classification algorithm by predefining the regions of interest (ROI) for PV, NPV, and BS. For each class, at least 50 ROIs were selected for training.

For OBIA, we used the feature extraction module in ENVI 5.5. In this method, an image is segmented into homogeneous areas based on two parameters: scale and merging level (spectral information). The scale parameter is unitless and controls the relative size of image objects (polygon or segment), with a smaller scale parameter resulting in more image objects. Merging combines adjacent segments with similar spectral attributes, a larger merging parameter results in more adjacent segments with similar colors and border sizes. Images were segmented at a 40–70 scale level and 10–30 merging level; choosing a high scale level results in fewer defined segments, while choosing a high merging level results in more segments to be aggregated into small segments within larger, textured areas. Specific parameter settings were adjusted interactively using the preview window in the feature extraction module because the texture and color features for individual images were dependent on the site-specific plant composition and background [6,26]. This also allowed us to predefine the training data for PV, NPV, and BS. The image was classified using the support vector machine (SVM) algorithm with all available attributes (spatial, spectral, and textural).

**Figure 2:**
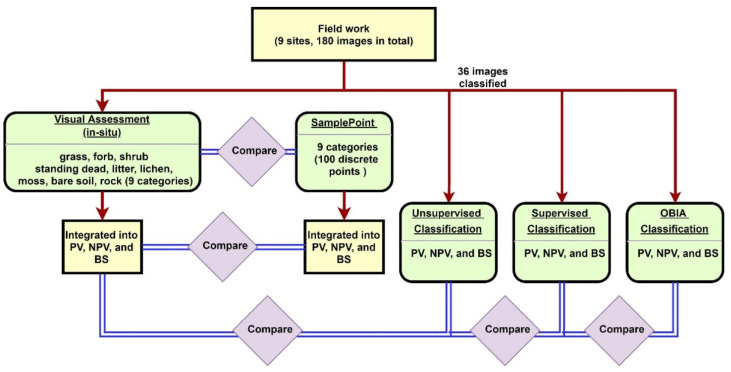
Methodology flowchart.

180 images (20 images per site for 9 sites) were used to compare the nine categories inventoried for both the field assessment and SamplePoint classification (Figure 2). We binned the nine categories into PV, NPV, and BS and compared results from both field and SamplePoint based on these bins. The unsupervised, supervised, and OBIA classification methods were applied to 36 selected images (4 images randomly per site) using PV, NPV, and BS categories. Results were compared to the field assessment.

Coefficient of determination (*R*^2^), root-mean-square error (RMSE) [41,42,43], and the Bland-Altman plot (Tukey mean difference plot) [44] were used to evaluate the comparison. The Bland-Altman plot allows the identification of any systematic differences between two measurements or possible outliers by plotting the differences between the two methods against their averages. The Cartesian coordinates of a given sample *S* with values *S*_1_ and *S*_2_ are:

(1) S(x,y)=((S1+S2)2 ,S1−S2)

The mean of *n* sample pairs’ difference (*S*_1-_*S*_2_) is the estimated bias, and the standard deviation (SD, σ) of the differences indicating the random fluctuations around this mean.

In the Bland-Altman plot, horizontal lines are drawn at the mean difference and at the limits of agreement. These are defined as the mean difference plus and minus 1.96 times the SD of the differences. The mean difference plus and minus 3.0 times the SD lines are defined as the extreme limits of agreement. A Wilcoxon test [45] of the paired samples was performed to compare paired data among the in situ assessments and four other methods (SamplePoint estimation, unsupervised classification, supervised classification, and OBIA). The Wilcoxon test was chosen to assess whether the population mean ranks differed among two applied FVC estimation methods. The paired Student’s t-test was not used because the data violate the normality distribution assumption [46]. In the paired samples Wilcoxon test, a *p*-value of 0.05 was selected as the threshold for significance.

## 3. Results and Discussion

### 3.1. Comparison of SamplePoint Estimation and In Situ Assessment

**Figure 3:**
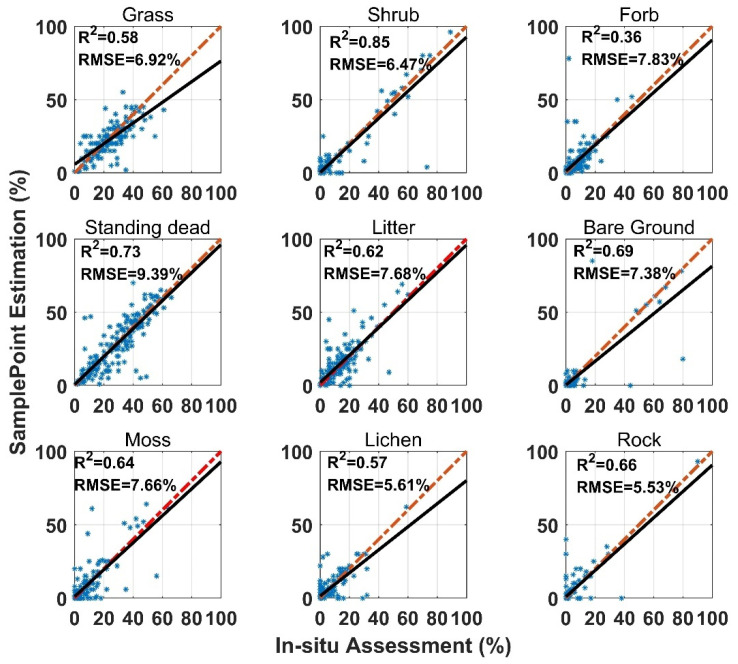
Comparison of in situ assessment and SamplePoint estimation for nine FVC categories (red dashed line is the 1:1 relationship; dark solid line is the linear regression). The regression significance level (*p*-value) is 0.01.

**Figure 4:**
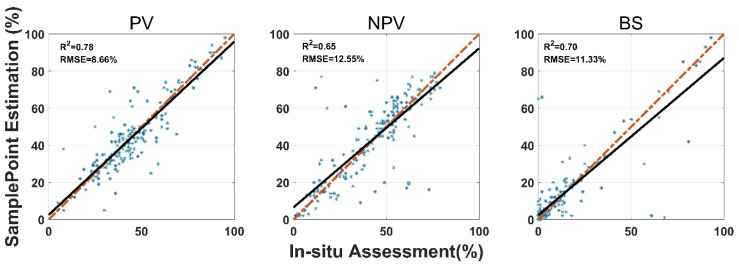
Comparison of in situ assessment and SamplePoint estimation for PV, NPV, and BS fraction cover (red dashed line is the 1:1 relationship; dark solid line is the linear regression). The regression significance level (*p*-value) is 0.01.

SamplePoint FVC estimation was consistent with the in situ assessment, however, the correlation coefficient (*R*^2^) varied among land surface-cover subcategories (Figure 3). Shrub and standing dead material had the highest *R*^2^ values (0.85 and 0.73), while forb and lichen had the lowest *R*^2^ values (0.36 and 0.57). Standing dead material had the highest RMSE (9.93%) (Figure 3). Shrub, standing dead material, and litter estimates from SamplePoint and in situ assessments were similar, as indicated by their regression and identity lines (Figure 3). Grass, bare ground, and lichen tend to be overestimated by SamplePoint when the fractional cover is larger than 20% (Figure 3). For the upscaling categories, the PV has the highest *R*^2^ (0.78), while the *R*^2^ values for NPV and the BS are 0.65 and 0.70, respectively (Figure 4).

**Figure 5:**
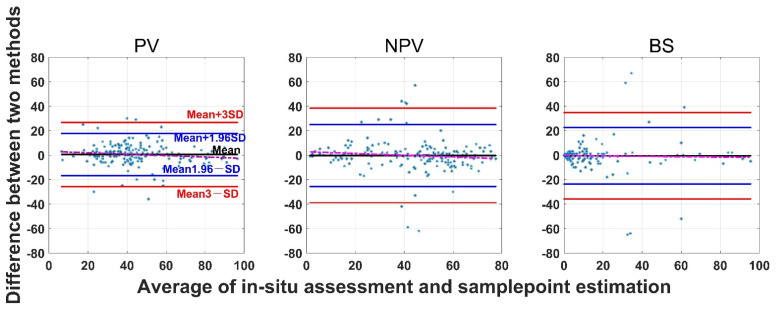
Bland-Altman plot of the difference between SamplePoint estimation and in situ assessment for PV, NPV, and BS fraction covers. Refer to the left panel for threshold values. The red dotted line is the regression between the two-method mean and the two-method difference.

The SamplePoint and in situ estimates of PV are close (Figure 4). The BS from SamplePoint tends to be overestimated above 25% of the fractional cover, compared with the in situ assessment (Figure 4). Similarly, NPV from SamplePoint is overestimated above 55% of the fractional cover and underestimated below 55% of the fractional cover (Figure 4). Most of the differences between the SamplePoint assessment and the in situ estimation of PV are within a ±3σ range, with several exceptions very close to the ±3σ threshold (Figure 5). This indicates that the SamplePoint and in situ methods are very similar. For the NPV and BS, almost all the differences are within the ±3σ range, although there are outliers that depart from the ±3σ range (Figure 5 and Table 1). These results are comparable to those reported in Booth et al. [47]. Since the theoretical basis of SamplePoint relies on a discrete classification for certain points (10 × 10 grids in this study) rather than on global image classification, there may be considerable bias for images in complex scenes. Like in situ assessment, which can be subjective, visual interpretation using SamplePoint software depends on the investigator experience.

**Figure 6:**
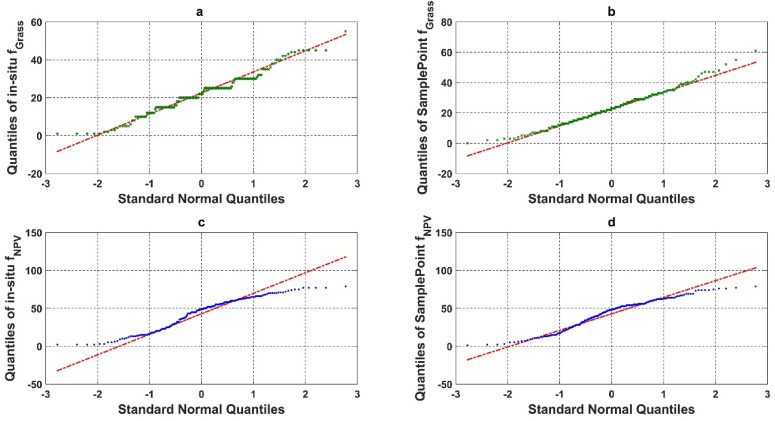
Quantile-quantile plot (qqplot) of (**a**) in situ assessment of Grass fractional cover (%), (**b**) SamplePoint classification of Grass fractional cover (%), (**c**) in situ assessment of NPV fractional cover (%), and (**d**) SamplePoint classification of NPV fractional cover (%).

We compared the quantile–quantile plot for the in situ assessment and SamplePoint classification of grass and NPV fractional cover (Figure 6). The in situ assessment of green grass cover based on the nine FVC categories had a clear clustering pattern (step curves) (Figure 6a). The piecewise polyline indicated that the in situ assessment had a categorical trend for the fractional cover estimation. This was caused by the protocol used in situ (as described in Section 2.2) as the fractional cover was estimated to the nearest 5% for values ranging from 10 to 90% and to the nearest 1% for values less than 10% and greater than 90% [39,40]. SamplePoint results resembled a normal distribution with a slight, right-skewed distribution (Figure 6b). This phenomenon indicated that SamplePoint can achieve a continuous estimate of detailed ground fractional cover, even when inputs are discrete points (10 × 10 grid in this study).

The in situ assessment of green grass cover based on the three-category schema (PV, NPV, and BS) showed no categorical trend but an under dispersed trend with negative excess kurtosis (Figure 6c). Comparatively, the qqplot from SamplePoint showed a slightly S-shaped curve, suggesting that it is approaching a normal distribution.

### 3.2. Comparison of Image Analysis Methods and In Situ Assessments

**Figure 7:**
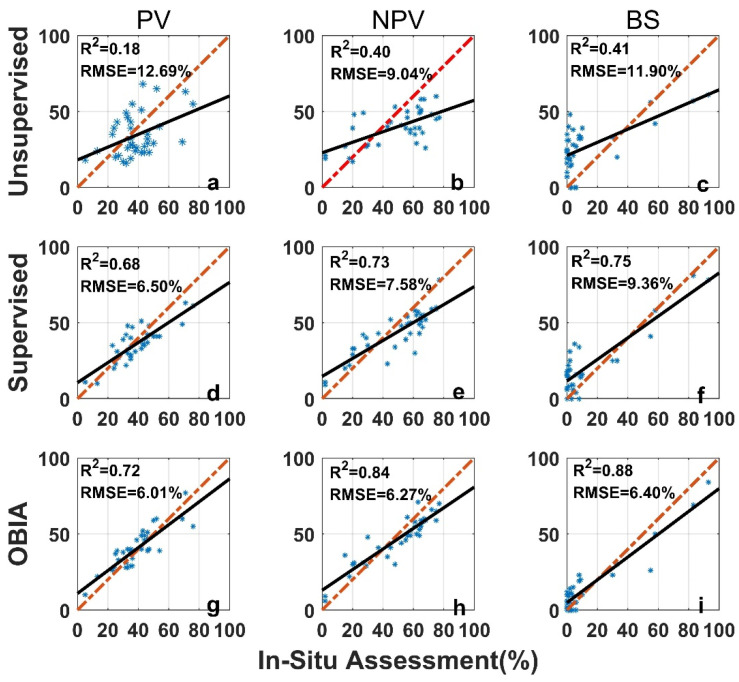
Comparison of in situ assessment and three image analysis methods (unsupervised (**a**–**c**), su-pervised (**d**–**f**), and OBIA (**g**–**i**)) for PV (**a**,**d**,**g**), NPV (**b**,**e**,**h**), and BS (**c**,**f**,**i**) fraction covers (the red dashed line is the 1:1 relationship and the dark solid line is the linear regression). The re-gression significance level (*p*-value) is 0.01.

**Figure 8:**
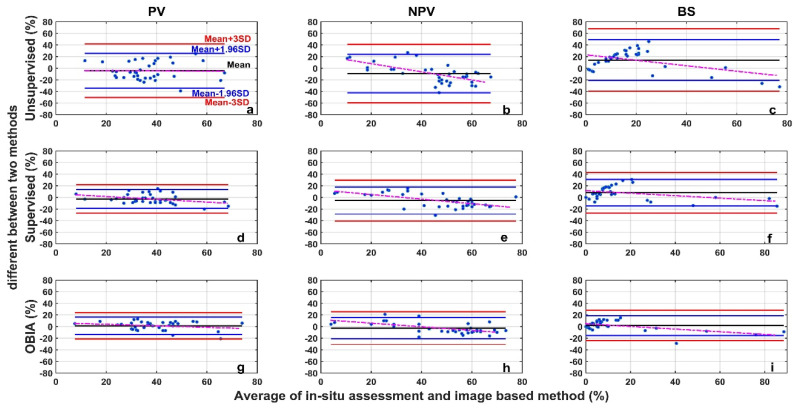
Bland-Altman plot of the difference between image analysis methods (unsupervised (**a**–**c**), supervised (**d**–**f**), and OBIA (**g**–**i**)) and in situ assessment for PV (**a**,**d**,**g**), NPV (**b**,**e**,**h**), and BS (**c**,**f**,**i**) fraction covers. Refer to the top-left panel for threshold values. The red dotted line is the regression between the two-method mean and the two-method difference.

The image analysis methods used in this study, including the unsupervised classification, the supervised classification, and the OBIA, perform differently than the in situ assessment (Figure 7). For all three categories (PV, NPV, and BS), the unsupervised image classification has the lowest *R*^2^ (all below 0.5) and the largest RMSE (Figure 7). The OBIA has the highest *R*^2^ (all above 0.7) and a relatively smaller RMSE (Figure 7). The supervised image classification performance is moderate. For the 36 images tested, the differences between image analysis methods and in situ estimates are within the ±3σ range (Figure 8), however, the range varies among methods (Table 1 and Figure 8). We found larger SDs (>15%) for unsupervised classifications (Table 1). Hence, ranges of ±1.96σ and ±3σ are smaller for the OBIA, moderate for the supervised method, and largest for the unsupervised method (Figure 8).

Mean differences for both PV and NPV were negative (Table 1) when we performed supervised and unsupervised classification analyses, indicating that these two methods underestimate PV and NPV, compared with the in situ assessment. In the OBIA, PV and BS are overestimated (mean differences > 0) whereas NPV is underestimated (Table 1).

**Figure 9:**
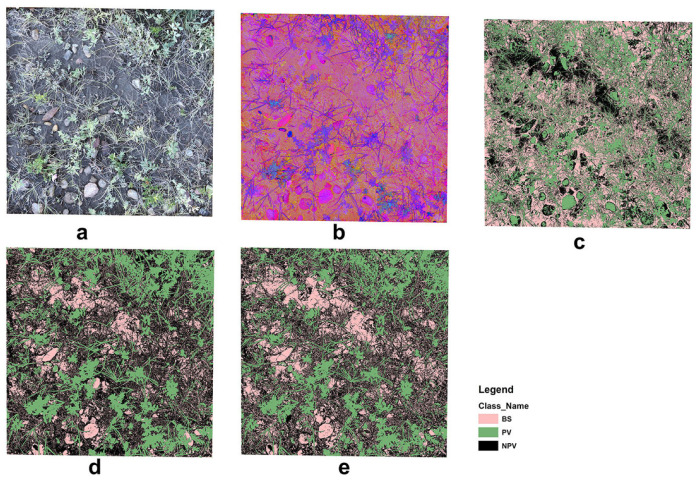
Example of image analyses: (**a**) original RGB image, (**b**) HIS image, (**c**) unsupervised image classification, (**d**) supervised image classification, and (**e**) OBIA.

The unsupervised classification misclassified rock, moss/lichen, and high reflectance regions in the background leading to biased estimations of PV, NPV, and BS fractional cover (Figure 9). The supervised classification was an improvement on the unsupervised method; however, the OBIA identified greater detail in the three fractional covers. Our findings are similar to those reported by Laliberte, Rango, Herrick, Fredrickson and Burkett [27], in which OBIA was also used to investigate the fractional cover of North American grassland. They suggested that shadow is the greatest problem in scene decomposition when applying OBIA to high-resolution, close-range digital photographs. This concern was also raised in Song, Mu, Yan and Huang [20], and was partially resolved by using a shadow-resistant algorithm. We did not include a shadow-resistant method to estimate the fractional cover in our study. However, we acknowledge that it is a problem for close-range photo processing, especially in heterogeneous grasslands with complex vertical structures and high biomass volumes. Shadow not only affects fractional cover estimation but also affects visual interpretation. This partially explains the outliers in our SamplePoint estimation (Figure 5), as our original photos, despite being umbrella-shaded, still had shadow effects.

### 3.3. Differences between In Situ Assessment, Visual Classification with SamplePoint Software, and Image Classification Methods

We performed a paired-samples Wilcoxon test between the in situ assessment and four remote methods to assess PV, NPV, and BS fractional covers. Measurements assessed using SamplePoint software and in situ samplings were not significantly different (threshold *p* = 0.05) among the three fractional covers, while assessment of BS was marginally insignificant (Table 2). NPV and BS estimated from the unsupervised classification, and the BS estimated from the supervised classification, were significantly different from the in situ assessment (Table 2). The OBIA assessment was not significantly different from the in situ assessment, however, *p* values of NPV and BS were close to the threshold (Table 2). Thus, SamplePoint assessment was the most consistent with in situ assessment, compared to unsupervised classification. Although the reliability of the OBIA would make it a suitable alternative for in situ methods, the OBIA requires sophisticated image processing and human training before it can be effective [19,27].

**Figure 10:**
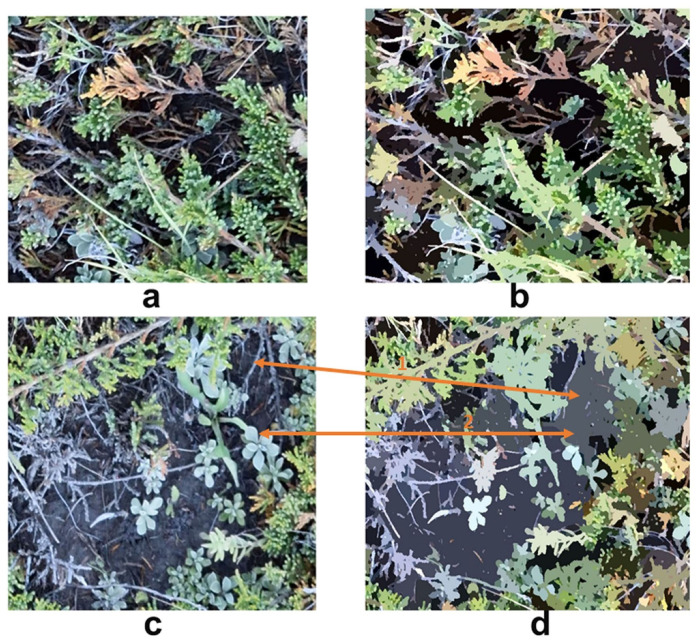
Comparison between the original image and OBIA region means: (**a**) original image (vegetation (grass and shrub) dominated), (**b**) OBIA region mean of (**a**), (**c**) original image (bare-soil dominated), and (**d**) OBIA region mean of (**c**). (**a**,**b**) are subsets of site EC2, plot E3 (shrub (*Juniperus*) dominated). The scale level was 50 and the merging level was 10.

We found that spatial scale and merging level had varying effects on OBIA analyses processing within a single image. The vegetation-dominated part of the image was accurately assessed for green vegetation, dead and senescent materials, and background (Figure 10a,b). This suggests the reason why the OBIA had greater accuracy than supervised and unsupervised classifications as it is based on relatively homogeneous segmented objects rather than pixels. In contrast, training samples selected in the supervised classification were largely based on polygons containing numerous mixed pixels [48]. However, in bare-soil dominated images, shrubs were incorrectly classified as background (Figure 10c,d, double arrow 1), as well as portions of green leaf (Figure 10c,d, double arrow 2). Because shrub branches, green leaf, dead materials, and bare soil (as well as moss, lichen, and rocks) all had different morphologies, a global setting of scale and merging level was unable to segment a heterogeneous scene [49,50].

**Figure 11:**
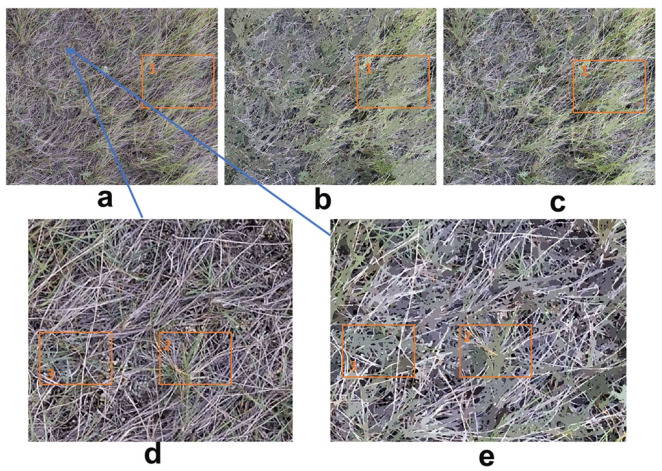
Illustration of spatial scale and merging-level effects on OBIA classification: (**a**) original image, (**b**) OBIA region mean scale was 60 and merging level was 10, (**c**) OBIA region mean scale was 40 and merging level was 5, (**d**) subset of (**a**), and (**e**) subset of (**b**). (**a**) was from site UG2, plot S5 (needle-and-thread-grass (*Hesperostipa comata*) dominated).

Different images with diverse species compositions required distinct scale and merging level settings when using OBIA (Figure 10 and Figure 11). Since juniper and needle-and-thread grass have different morphologies and community structure, the scale and merging level were 50 and 10 for the site EC2, plot E3, and 40 and 5 for the site UG2, plot S5. The latter image had greater fragmentation with layers of green grass (top), senescent grass (middle), and dead material (bottom). However, the scene was relatively simple in the former image except for the misclassification of shrub branches.

We tested the effect of spatial scale and merging level on OBIA classification (Figure 11). Larger scale and merging levels (60 and 10) caused misclassification of green grass (Figure 11b), while the smaller scale and merging levels (40 and 5) had better results (orange rectangle 1 in Figure 11a–c). A scaled-in view (as shown in Figure 11d,e (orange rectangle 2 and 3)) resulted in the pseudo-enlargement of green grass objects. This indicated that the selection of proper segmentation and merging parameters related to scene composition was critical for accurate assessment using OBIA. As mentioned above, we used the preview window in the Feature Extraction Module of ENVI 5.5 for the interactive adjustment of these parameters. This is time-consuming and needs knowledge of OBIA.

As is apparent in these comparisons, the SamplePoint-processed results were highly related to in situ estimation, even with nine different categories (Figure 3). The unsupervised image classification method was unable to discriminate PV, NPV, and BS with the desired accuracy, while supervised image classification outperformed the unsupervised method. OBIA had the highest accuracy among the three image classification methods compared with the in situ estimation.

## 4. Conclusions

In this study, a mobile camera stand equipped with a NIKON D5500 camera was used to photograph vegetation plots in a typical northern mixed grassland, the Grassland National Park, Canada. This grassland type has a large amount of dead senescent vegetation material. The imagery was processed by SamplePoint, unsupervised, supervised, and object-based image classification approaches to derive the vegetation fractional covers, which were compared with in situ visual assessment.

Our results demonstrated that imagery processing methods for mixed grassland vegetation communities can accurately determine the fractional vegetation cover in sample plots, which is comparable to in situ measurement. We found that SamplePoint software estimates corresponded highly to in situ assessments, accurately distinguishing and quantifying PV, NPV, and BS fractional covers as well as the detailed vegetation community categories. The object-based image analysis method performed better than the unsupervised and supervised classification methods and produced reasonable coefficients of determination (>0.7) for PV, NPV, and BS, comparable to in situ assessment. The OBIA method nevertheless required sophisticated processing knowledge. Meanwhile, the unsupervised classification method lacked accuracy in the discrimination of fractional cover in mixed grassland plots. These results suggest that the in situ estimation method is comparable with a more accurate SamplePoint approach based purely on imagery. Further research into image-based estimation approaches could resolve ongoing issues with shadow and various image-scene compositions.

## Figures and Tables

**Table 1:** Mean, standard deviation (SD), and mean ± 1.96 (or 3) SD of the differences between in situ assessment and four imagery methods (SamplePoint estimation, unsupervised classification, supervised classification, and OBIA).

	SamplePoint			Unsupervised Classification			Supervised Classification			OBIA		
	PV	NPV	BS	PV	NPV	BS	PV	NPV	BS	PV	NPV	BS
Mean (%)	0.45	−0.37	−0.52	−4.39	−9.22	14.06	−2.64	−5.36	8.08	1.31	−2.75	1.75
SD (%)	8.76	12.90	11.78	15.35	16.83	17.92	8.22	11.70	11.65	7.59	9.41	8.72
Mean ± 1.96SD	−16.7 ~ +17.6	−25.7 ~ +24.9	−23.6 ~ +22.6	−34.5 ~ +25.7	−42.2 ~ +23.8	−21.1 ~ +49.2	−18.8 ~ +13.5	−28.3 ~ +17.6	−14.8 ~ +30.9	−13.6 ~ +16.2	−21.2 ~ +15.7	−15.3 ~ +18.8
Mean ± 3SD	−25.8 ~ +26.7	−39.1 ~ +38.3	−35.9 ~ +34.8	−50.4 ~ +41.7	−59.7 ~ +44.5	−39.7 ~ +67.8	−27.3 ~ +22.0	−40.5 ~ +29.7	−26.9 ~ +43.0	−21.5 ~ +24.1	−31.0 ~ +25.5	−24.4 ~ +27.9

**Table 2:** *p*-value of the paired-samples Wilcoxon test between in situ assessment and four other approaches (SamplePoint estimation, unsupervised classification, supervised classification, and object-based image analysis (OBIA)).

	SamplePoint ^1^			Unsupervised Classification ^2^			Supervised Classification ^2^			OBIA ^2^		
	PV	NPV	BS	PV	NPV	BS	PV	NPV	BS	PV	NPV	BS
*p*-value (%)	0.25	0.33	0.071	0.11	0.003 *	0.0002 *	0.08	0.10	0.0005 *	0.17	0.06	0.089

^1^*n* = 180; ^2^ *n* = 36. * indicates *p*-value < 0.05

## Data Availability

Data available on request due to restrictions.
